# Audit of Clinical Care Received by COVID-19 Patients Treated at a Tertiary Care Hospital of Nepal in 2021

**DOI:** 10.3390/tropicalmed7110381

**Published:** 2022-11-16

**Authors:** Shrawan Kumar Mandal, Jenish Neupane, Ajay M. V. Kumar, Hayk Davtyan, Pruthu Thekkur, Anup Jayaram, Bimal Sharma Chalise, Manisha Rawal, Manu Paudel, Bishwodip Baral, Rajesh Kumar Shah, Kijan Maharjan, Sanjay Shrestha, Lilanath Bhandari, Nisha K.C., Nabaraj Gautam, Avinash K. Sunny, Nishant Thakur, Koshal Chandra Subeedee, Sushil Kumar Mandal, Anup Bastola

**Affiliations:** 1 Sukraraj Tropical and Infectious Disease Hospital, Ministry of Health and Population, Government of Nepal, Kathmandu 44600, Nepal; 2 International Union Against Tuberculosis and Lung Disease, 2 Rue Jean Lantier, 75001 Paris, France; 3 International Union Against Tuberculosis and Lung Disease, South-East Asia Office, C-6 Qutub Institutional Area, New Delhi 110016, The National Capital Territory of Delhi, India; 4 Yenepoya Medical College, Yenepoya (Deemed to be University), University Road, Deralakatte, Mangaluru 575018, Karnataka, India; 5 Tuberculosis Research and Prevention Center, NGO, Yerevan 0014, Armenia; 6 Manipal Institute of Virology, Manipal, Academy of Higher Education, Manipal, Udupi 576104, Karnataka, India; 7 WHO Country Office, Kathmandu 44600, Nepal; 8 Epidemiology and Disease Control Division, Kathmandu 44600, Nepal; 9 Tribhuvan University Teaching Hospital, Maharajgunj, Kathmandu 44600, Nepal

**Keywords:** COVID-19, Nepal, clinical care, outcomes, operational research, SORT IT

## Abstract

Like the world over, Nepal was also hard hit by the second wave of COVID-19. We audited the clinical care provided to COVID-19 patients admitted from April to June 2021 in a tertiary care hospital of Nepal. This was a cohort study using routinely collected hospital data. There were 620 patients, and most (458, 74%) had severe illness. The majority (600, 97%) of the patients were eligible for admission as per national guidelines. Laboratory tests helping to predict the outcome of COVID-19, such as D-dimer and C-reactive protein, were missing in about 25% of patients. Nearly all (>95%) patients with severe disease received corticosteroids, anticoagulants and oxygen. The use of remdesivir was low (22%). About 70% of the patients received antibiotics. Hospital exit outcomes of most (>95%) patients with mild and moderate illness were favorable (alive and discharged). Among patients with severe illness, about 25% died and 4% were critically ill, needing further referral. This is the first study from Nepal to audit and document COVID-19 clinical care provision in a tertiary care hospital, thus filling the evidence gap in this area from resource-limited settings. Adherence to admission guidelines was excellent. Laboratory testing, access to essential drugs and data management needs to be improved.

## 1. Introduction

Coronavirus disease 2019 (COVID-19) is a viral infection caused by severe acute respiratory syndrome coronavirus 2 (SARS-CoV-2). This virus was first identified in December 2019 in the Wuhan, Hubei province of China [1]. It became a cause of an unprecedented pandemic within a few months and is still creating havoc in the entire world.

There were around 466 million confirmed cases of COVID-19 reported globally as of March 2022, and of them, 6 million (1.3%) had died. For the same period, Nepal had reported a total of 1,118,197 confirmed cases, and among them, 11,950 (1.06%) had died. The surge of cases reported as a second wave of COVID-19 in Nepal began in March 2021 with the highest number of cases reported mid-May 2021 when the number of confirmed cases per day was more than 9300. This number then dropped in a steady manner, reaching around 1000 cases per day by early September 2021. By March 2022, only 10 confirmed cases were identified [2,3].

The pandemic has not only overwhelmed the healthcare systems but also altered the socioeconomic balance of nations. The impact is much more catastrophic in low-income countries such as Nepal. Fight against the disease in Nepal was not effective due to lack of access to diagnostic tools, preventive and therapeutic measures, and the results have been devastating [4,5]. Even though highly effective COVID-19 vaccines were developed after the first wave of COVID-19, given the mismatch between the demand and supply, it was extremely hard to vaccinate everybody within the short period of time. Vaccination has picked up pace in recent times with 18.7 million people receiving at least one dose of vaccine as of March 2022 [2].

Nepal has a fragile healthcare system which was overwhelmed by the large number of patients that required hospital care during the second wave of COVID-19. The limited availability of resources such as Intensive Care Units, ventilators, oxygen and other vital medicines and equipment had caused difficulties in care provision [6].

There have been several published research studies from various countries on COVID-19 and the factors associated with poor outcomes in the past two years. To summarize, the following risk factors have been identified: advanced age, male sex, people with comorbidities such as hypertension, diabetes mellitus, obesity, malignancy, chronic kidney disease, cardiovascular and cerebrovascular disease and history of smoking [7,8,9]. Hypoxia and the presence of other respiratory symptoms (shortness of breath, chest pain) and gastrointestinal symptoms (nausea, vomiting and abdominal pain) are more likely to progress to severe disease, and the presence of pneumonia and end-organ disease has been associated with higher mortality [9,10,11]. Laboratory parameters that were associated with poor outcomes include lower lymphocyte count, lower platelet count, elevated creatinine and the presence of higher levels of biomarkers in the blood such as D-Dimer, C-reactive protein and procalcitonin [10,11,12]. On the other hand, receipt of the COVID vaccine is associated with substantially reduced risk against adverse outcomes of COVID-19 [13]. A previous study from Nepal that was conducted during the first wave of the COVID-19 pandemic reported that about 6% of the admitted patients died and those who died had risk factors similar to ones described above [14]. Although there has been much evidence on patient outcomes and factors associated with poor outcomes [8], there is not much information or description of the COVID care received by the patients in programmatic settings of low- and middle-income countries (LMICs). Evidence exists from high-income countries that there is a need for having well-defined clinical workflow to ensure care quality for COVID-19 patients [15]. Although the hospitals in low- and middle-income countries have standard operating procedures (SOPs) for admission and COVID care, the facilities are usually challenged to offer optimal care as per the SOPs, given the programmatic realities. It is imperative to audit the COVID care because understanding the gaps in care provision and fixing them has the potential to improve the treatment outcomes. A clinical audit is a great tool to compare the current clinical practice with the recommended guidelines. There has not been any study from Nepal auditing the COVID care. Such evidence may also help in strengthening the preparedness of the hospital during future pandemics in areas such as triaging, admission, testing, treatment, recording and reporting. Hence, we planned this operational research with the following aim and objectives.

The overall aim of the study was to audit the clinical care received by the COVID-19 patients admitted in Sukraraj Tropical and Infectious Disease Hospital (STIDH), which is a tertiary care hospital in Nepal designated for COVID-19 care, and describe the hospital outcomes. Specific objectives were to assess and describe: (i) the proportion eligible for admission as per national guidelines for clinical care in the healthcare facility; (ii) the proportion who received laboratory investigations and treatments for clinical care in the healthcare facilities; and (iii) the hospital exit outcomes (discharge, discharge on request, death, referral).

## 2. Materials and Methods

### 2.1. Study Design

This was a cohort study involving the secondary analysis of routinely collected hospital data.

### 2.2. Setting

#### 2.2.1. General Setting

The Federal Democratic Republic of Nepal is a landlocked country in Southeast Asia with an estimated population of 30 million. It borders China in the north and India in the south, east, and west. It has seven provinces and 77 districts [16,17].

#### 2.2.2. Specific Setting

The study was conducted at STIDH. This is the central infectious and tropical disease hospital established in 1933 and located in Kathmandu, Nepal. It is a 100-beded national referral hospital. It also provides training to undergraduate and postgraduate medical students. Students from different countries undergo training in infectious and tropical diseases in this hospital. The hospital has a well-equipped laboratory with pathologists, microbiologists, radiologists, laboratory assistants and other support staffs. It provides both in-patient and outpatient services and has an emergency department (ED), a high-dependency unit (HDU) and an intensive care unit (ICU). On average, about 500 patients from all over Nepal visit the OPD (outpatient department) every day. Similarly, about 50 patients per day are provided service in the emergency department.

This was designated as the COVID-19 central hospital by the Government of Nepal in 2020. Several infrastructural modifications were made to the hospital for providing COVID-19 services. Extra beds were added to increase the capacity, and a strict isolation room, COVID HDU and ICU were added. Additionally, the capacity of performing all relevant diagnostic tests and examinations was introduced for COVID-19 including reverse transcription polymerase chain reaction (RT-PCR) tests, high-resolution computed tomography (HRCT), and computed tomography pulmonary angiogram (CTPA), among other tests.

#### 2.2.3. COVID-19 Management in STIDH

COVID-19 patients and suspected cases from all over Nepal visit STIDH to receive diagnostic and therapeutic services. People with symptoms of COVID-19, close contacts of COVID-19 positive cases and those with travel history (those who entered Nepal recently) were tested with an antigen test and RT-PCR for the diagnosis. They were kept under observation, if medical attention is required (those who are under risk of deterioration of symptoms based on their vitals). Otherwise, they were sent with advice to stay in quarantine until the test results are known.

Patients with confirmed COVID-19 infection by either RT-PCR or antigen test undergo triage and were classified as mild, moderate, severe and critical according to clinical criteria recommended in Clinical management of COVID-19: living guidance version 1.4 published by WHO on 25 January 2021 [18]. Mild cases included those who were symptomatic but had no signs of viral pneumonia or hypoxia. Moderate cases included patients having clinical signs of pneumonia (dyspnea, fast breathing) as per clinical judgement but no signs of severe pneumonia, including SpO2 ≥ 90% on room air. Severe cases included those cases with clinical signs of pneumonia (fever, cough, dyspnea) along with one of the following: respiratory rate > 30 breaths/min; severe respiratory distress; or SpO2 < 90% on room air. Most of the mild and asymptomatic cases were treated on an outpatient basis and sent home after providing medications, advice on home isolation and explanation of danger signs and symptoms. They were advised to visit a hospital if any of the danger signs such as visible respiratory distress or the appearance of new symptoms emerge. Mild cases with comorbidities or moderate to severe cases were admitted and kept under observation. All necessary and available blood tests and radiological investigations were performed.

For mild COVID-19 cases, no specific laboratory tests were recommended, while for moderate, severe or critical COVID-19 cases, the following tests were recommended: complete blood count and differential count, renal function and electrolyte tests liver function tests and if available, tests can be sent for D-dimer level, lactate dehydrogenase level, quantitative C-reactive protein, troponin, ferritin and procalcitonin. Samples were to be sent for cultures of blood, sputum, and, if indicated, urine, before starting antibiotics for any reason or if bacterial sepsis is suspected [19]. Severe cases were managed on priority to stabilize the vitals by providing necessary emergency drugs. Intubation was done when required. Further care was monitored by a critical care specialist and physician. Treatment included oxygen, antipyretics, antitussives, corticosteroids and anticoagulants. In the admitted patients, remdesivir and convalescent plasma therapy were also administered according to the WHO living guideline for therapeutics of COVID-19 [20] and after consultation with patients and their relatives. According to the oxygen demand (oxygen saturation below 94% or having difficulty in breathing) and clinical status of the patient, oxygen was given via various devices, non-invasive ventilation and mechanical intubation.

There was a strong recommendation for systemic corticosteroids in patients with severe and critical COVID-19 [18]. It was advised that treatment decisions with antiviral drugs including remdesivir should be made by the healthcare provider based on their discussion with the patient and their legal guardians. Given the emerging evidence of potential benefits of Remdesivir, it was considered in moderate and severe patients [19].

During the early days of the pandemic, patients were not discharged until they tested negative for COVID-19 via RT-PCR. Later, due to the increased load and limited capacity, patients were discharged after stabilization, which was determined by improvement in clinical status (no distress due to the symptoms), improving vitals and no/less requirement for oxygen. Mild cases were sent back home straight from the emergency department without admitting them and without creating patient file for them. Occasionally, patients were referred to other specialized centers in case of complications when there was a need for specialized medical care not available in STIDH such as the case of cardiovascular complication, need for dialysis and neurological complications. The guidelines on admission, laboratory investigations and medications stratified by severity of the illness is summarized in Table 1.

#### 2.2.4. Recording of Data

The hospital admission files (paper form) containing all treatment-related information were kept for each admitted patient. A part (administrative information, demographics, diagnosis and outcome) of this information was then transferred to the Information Management Unit (IMU) Nepal software, the official portal from the Information Management Unit of Nepal Ministry of Health and Population.

#### 2.2.5. Guidelines for Management of Care

During the initial period of the COVID-19 pandemic, there were a lot of uncertainties and little scientific evidence and guidelines to be followed for the clinical care of COVID-19 patients. We compared the care with the clinical management of COVID-19 Living guidance by WHO updated on 25 January 2021 [18] and also the interim clinical guidance for care of patients with COVID-19 in healthcare settings by Nepal Medical Council update 1 published on June 2020 [19].

### 2.3. Study Population

The study population included all confirmed COVID-19 patients admitted to STIDH during the period of April to September 2021 (covering most of the second wave in the country). Since all the patients within the study period were included, there was no sampling strategy. We did not calculate sample size because this was not a pre-designed survey with a pre-defined reference population.

### 2.4. Data Variables and Sources

Data were obtained from the hospital records, including hospital patient database, paper based records (register) and the patient file. The data in the patient file (primary source) was considered as final in case of discrepancies. The variables included demographic variables, clinical symptoms, presence of comorbidity, laboratory parameters, treatments provided, vaccination status and hospital exit outcomes.

### 2.5. Data Management and Analysis

The data were collected in Microsoft Excel format. This was cross-checked with the data in the paper-based sources and verified. We analyzed the data using EpiData Analysis (v2.2.2.187, EpiData Association, Odense, Denmark) and Stata software (v12, Statacorp, College Station, TX, USA).

We described the demographic and clinical profile of study participants by using frequencies and proportions (for categorical variables) and mean (standard deviation (SD)) or median (interquartile range) for continuous variables, as appropriate. The frequencies and proportions were used to summarize the uptake of essential investigations and abnormal values among the study participants. Similarly, the frequency and proportions were used to describe the medicines used among the study participants during their stay in the hospital. There were four possible hospital exit outcomes—discharge, discharged on patient’s request (against medical advice), death and referral to higher level of care.

## 3. Results

### 3.1. Eligibility of Patients for Admission and Their Socio-Demographic/Clinical Characteristics

**Figure 1:**
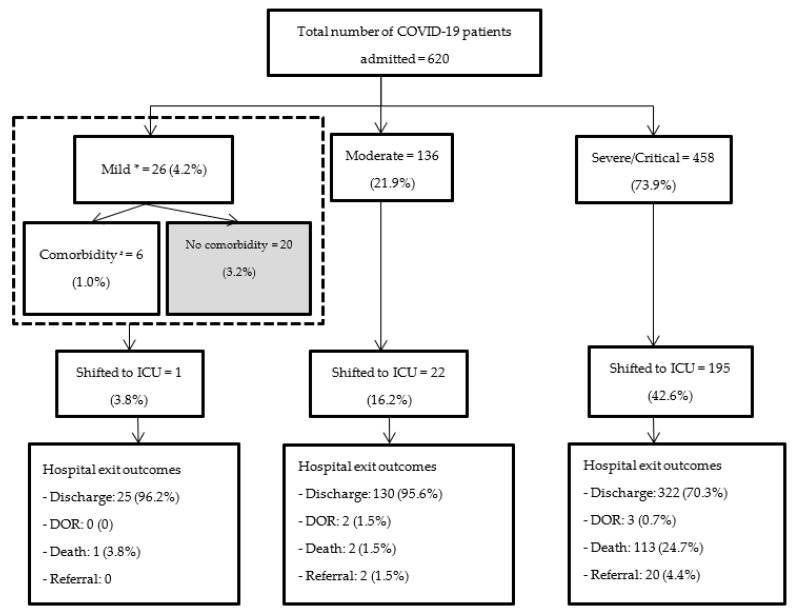
Severity at admission, shift to ICU and hospital exit outcomes among COVID-19 patients admitted in a tertiary teaching hospital of Nepal during April to September 2021. * Severity is categorized according to WHO clinical living guideline; ^#^ Comorbidities include diabetes, hypertension, chronic kidney disease, hypothyroidism, known cardiac disease; Except for mild cases without comorbidity, all the others were eligible for admission. Abbreviations: ICU—Intensive Care Unit; DOR—Discharge on request.

There were a total of 640 COVID-19 patients admitted during the study period. Of them, only 620 (97%) had patient files and were included in the analysis. The eligibility of these patients for admission and outcomes of the patients, stratified by severity of illness, is depicted in Figure 1. About 600 (97%) of patients were eligible for admission.

Of the admitted patients, 397 (64%) were males. The mean (SD) age was 51.3 (15.8) years. Nearly three-fourths of the patients had severe illness. Cough and fever were the predominant symptoms in people with mild and moderate illness, whereas cough and shortness of breath were the predominant symptoms in people with severe illness. The majority (380, 61%) of the patients had no comorbidities. The most common comorbidities were hypertension (151, 24%) and diabetes (95, 15%). A total of 352 (55%) patients had oxygen saturation of 85% or less at the time of admission and 532 (85%) patients had not received vaccination against COVID-19 (Table 2).

### 3.2. Management of COVID-19 Patients

Although all the individuals with severe form of illness were expected to receive all the investigations listed in Table 3, except for blood sugar, there were gaps in the conduct of other investigations. In people with severe illness, D-dimer was conducted in 368 (80.3%) patients, C-reactive protein in 31 (67.9%) patients, blood culture in 162 (35.4%) and sputum culture in 168 (36.7%) patients. Similarly, in individuals with moderate illness, D-dimer was conducted in only 87 (64%) patients, while C-reactive protein was conducted in 96 (70.6%) patients, although they were eligible. In contrast, patients with mild illness who were not eligible for any investigations had D-dimer (19, 73.1%) and C-reactive protein (23, 88.5%) tests completed.

Among all the patients, the most common abnormality in the laboratory investigation was an elevated C-reactive protein found in 384 (89%) of the tested patients followed by an elevated D-dimer in 254 (54%) of the patients. At least one microorganism was isolated from 48 (25%) patients in their sputum and from 10 (6%) patients in their blood sample (Table 3).

All individuals with severe illness who were eligible to receive corticosteroids and anticoagulant agent had received it. However, even though all individuals with severe illness were eligible to receive remdesivir, only 116 (25.3%) received it. Although all the patients with moderate illness were supposed to receive anticoagulants, only 106 (77.9%) had received it. Individuals with moderate illness at presentation who later received corticosteroids and remdesivir during the course of treatment were 101 (74.3%) and 19 (14.0%), respectively. Similar, the use of remdesivir (1, 3.8%), corticosteroids (8, 30.8%) and anticoagulant (11, 42.3%) was seen among patients with a mild form of illness at their presentation. In addition, 6 (23.1%) patients with mild illness and 43 (31.6%) patients with moderate illness had received antibiotics later on during the course of treatment. Similarly, 4 (15.4%) patients with mild illness and 107 (78.7%) patients with moderate illness later on received oxygen (Table 4).

### 3.3. Hospital Exit Outcomes

Most (157, 95%) of the patients with mild and moderate illness were alive and discharged. Among patients with severe illness, about 113 (25%) died during the hospital stay and 20 (4%) had to be referred to a higher level of care for further care.

## 4. Discussion

This is the first study in Nepal to audit and document COVID-19 clinical care provision in a tertiary care hospital and addresses the gap in this topic from LMICs such as Nepal. There were some noteworthy and interesting findings.

First, about 97% of the admitted patients were in fact eligible for admission as per national guidelines. Only 3% of patients (mild cases without any comorbidities) who did not fulfill the criteria were admitted. This adherence to guidelines is commendable, especially so, in light of the fact that the study period overlapped with the most dreadful second wave of COVID-19 in Nepal with a huge surge in the number of cases. Unfortunately, our study did not investigate reasons for admitting ineligible patients. We also did not have information on numbers of eligible patients who were not admitted—it might be possible that some eligible patients missed admission due to the overloading of hospital capacity. Future research is required to explore these aspects. However, we suspect that at the time of admission, these cases may have been admitted closer to the end of the second wave of COVID/study period when the number of daily cases in the country went down and hospital capacity allowed the admission of such cases without risk of refusing treatment to those who were eligible. As expected, during the peak of the COVID wave when the number of cases was surging, the majority of patients (about three out of four patients) had severe illness. Similar to the previous studies, male patients were predominant, and the most common age group was 40–59 years. Nearly one-third of patients who were admitted had comorbidities [21]. The most common symptoms in the patients were cough, fever and shortness of breath, with the latter one being more prevalent in severe patients. This finding was similar to the previous studies [11,22].

Second, most of the patients underwent baseline laboratory investigations, but some specific tests that help to predict the potential outcome of the disease, such as D-dimer and C-reactive protein, were missing in about one-fourth of the patients [12]. The reasons for not performing these tests were not a part of our study; however, based on the observation during clinical practice, we speculate that tests in some of these patients were completed, but they were not recorded properly in the patient files. Additionally, some errors may have occurred when ordering the laboratory tests, and these tests were not requested due to human error. Another reason could be the high number of requests and insufficient laboratory capacity. The other problem was that many patients with mild illness who were not eligible to receive the tests received them—indicating that the limited resources were not efficiently used.

In terms of medications, nearly all the patients with severe disease received steroids and anticoagulant therapy, as expected. The use of remdesivir was relatively low, which was primarily because of the unavailability of the medicine. The low use of remdesivir cannot be labeled as lack of proper care, given the uncertainties prevalent regarding its use at the time. Some of the patients with mild/moderate illness also received remdesivir, although this was not indicated by the guidelines. Some of the patients with mild/moderate illness also received remdesivir, although this was not indicated by the guidelines. A potential reason for this could be related to the quick progression of illness from mild/moderate to severe/critical during the course of hospitalization, which was not documented.

About 70% of the patients received antibiotics, even though only about one-third of the patients were tested for culture (indicating suspicion of bacterial secondary infection) and only about 25% of those tested were culture positive. The unnecessary and overuse of antibiotics, such as here, has the potential to increase the risk of development of antimicrobial resistance and should be minimized. This finding is similar to that in other settings. A meta-analysis by Langford et al. demonstrated that around 75% of all COVID-19 patients across the world received antibiotics, and for most of them, it was potentially unnecessary [23]. Oxygen was provided to every nine out of ten patients. Almost everyone received it with a non-invasive mode, and only 1% of patients were intubated. Current recommendations for the management of COVID-19 cases in terms of oxygen support are highlighting the importance of non-invasive methodologies as well as the timely intubation in patients with aggravating disease, mental status, and the development of respiratory acidosis in patients with acute respiratory distress syndrome. Delayed intubation is associated with unfavorable treatment outcomes [24]. It is hard to assess if oxygen provision methods and the timing in our study was appropriate, as our study were not designed to evaluate this.

Third, nearly one in four patients with severe illness had fatal outcomes, while in mild and moderate patients, this outcome was only 2%. These findings are consistent with previous studies [21,25].

The strengths of the study included the use of routine data from a tertiary-level hospital covering the period of the second wave of the COVID-19 in Nepal, thus reflecting ground realities. Additionally, we reported the study findings in compliance with the Strengthening the Reporting of Observational Studies in Epidemiology (STROBE) statement [26]. The study had some limitations as well. This was a retrospective study based on the hospital record of a single center, and we did not have control over the original data collection (patient files, etc.). There were missing data in some crucial parameters such as those related to the laboratory investigation, treatments received, vaccine doses, timing of vaccine receipt and eventual outcomes of patients referred to higher center. Only data at the time of admission were available, but we did not have (temporal) data on disease progression. This might explain why some patients with mild/moderate illness at baseline received investigations and medications reserved for severe illness. The data of many patients who visited the hospital but were not admitted were not available as well. Some laboratory investigations that were completed privately outside the hospital before admission might not have been recorded. As a result of all of these limitations, we could not perform a robust analysis of possible associations of these factors with unfavorable outcomes among COVID-19 patients. Since this is a study from a single hospital with no pre-defined reference population, we are unable to generalize the findings beyond the study setting.

Despite the limitations, our study has some operational implications. First, guidelines in assessing the eligibility of admitted patients were followed in most of the patients. There were only a few cases of incorrect admission. Due to the huge and sudden overload of the patients in the hospital, patients who were eligible for admission but could not be admitted were not recorded properly. Admission eligibility guidelines need to be strictly enforced to ensure that there is no refusal of services to those in most need as well as to ensure the correct use of resources in a limited resource setting. Second, laboratory tests for markers predicting disease outcomes (such as D-dimer and C reactive protein) need to be ordered for all eligible patients for the proper planning of care and timely intervention. Additionally, the recording system of the hospital may require upgrading (potentially to an electronic database) to ensure the accuracy of data and make data-driven decision making in terms of care provision possible. Third, oxygen provision algorithms and timing need to be assessed in the hospital with a separate research study to ensure correct use of the oxygen therapy. Fourth, the treatment protocol should be enforced strictly to limit the use of antibiotics unless necessary. Timely advocacy is required to ensure the availability of essential medications such as remdesivir, tocilizumab and other newer drugs. Finally, a huge gap in vaccination in the admitted patients calls for an immediate intervention to increase the vaccination coverage of the general population. Unlike vaccine hesitancy and acceptance issues in other settings, the key problem in Nepal was the unavailability of vaccines to meet the demand during the study period. Thus, only high-risk groups (such as elderly and those with comorbidities) were prioritized by the Government of Nepal in the early stages of the pandemic.

## 5. Conclusions

We studied clinical care of the COVID-19 in a tertiary care hospital setting and identified several aspects for improving the care provision. Adherence to admission guidelines was good, but it can be improved. Gaps in laboratory testing and access to treatment were observed. There were several deficiencies in the recording and reporting of data—this calls for action to improve the data management system of the hospital.

## Figures and Tables

**Table 1:** Guidelines on admission, laboratory investigations and medications (stratified by severity of the COVID-19 illness) followed in a tertiary hospital of Nepal during April to September 2021.

Parameter	Mild	Moderate	Severe
**Admission**	N/Y *	Y	Y
			
**Laboratory investigations**			
Hemoglobin	N	Y	Y
Total leucocyte count	N	Y	Y
Platelet count	N	Y	Y
D-dimer	N	Y	Y
C-reactive protein	N	Y	Y
Random blood sugar	N	Y	Y
Serum creatinine	N	Y	Y
Blood culture	N	N	Y
Sputum culture	N	N	Y
			
**Medication**			
Remdesivir	N	N	Y
Corticosteroids	N	N	Y
Anticoagulants	N	Y	Y
Antibiotics	N	N	Y/N **
			
**Oxygen support**			
Invasive mechanical ventilation	N	N	Y/N ^
Non-invasive ventilation	N	N	Y/N ^
Low-flow O2 device	N	N	Y

Note: N means ‘No’—particular service was not applicable for the patient; Y means ‘Yes’—the defined service was applicable for the patient; * Mild cases were eligible for admission if they had other comorbidities; ** Antibiotics are indicated only if there is a suspicion of a secondary bacterial infection; ^ For oxygen support, first, a low-flow O2 device will be used. If there is no improvement in oxygen saturation, non-invasive ventilation and invasive mechanical ventilation will be considered.

**Table 2:** Baseline sociodemographic and clinical characteristics of COVID-19 patients (stratified by severity of illness) admitted in a tertiary hospital of Nepal from April to September 2021.

Characteristics	Mild ^		Moderate ^		Severe/Critical ^		Total	
	n	(%) *	n	(%) *	n	(%) *	n	(%) *
Total	26	(100)	136	(100)	458	(100)	620	(100)
**Age (in years)**								
≤19	2	(7.7)	3	(2.2)	0	(0)	5	(0.8)
20–39	12	(46.2)	53	(39.0)	80	(17.5)	145	(23.4)
40–59	9	(34.6)	49	(36.0)	216	(47.2)	274	(44.2)
≥60	3	(11.5)	31	(22.8)	162	(35.4)	196	(31.6)
**Sex**								
Male	16	(61.5)	87	(64.0)	294	(64.2)	397	(64.0)
Female	10	(38.5)	49	(36.0)	164	(35.8)	223	(36.0)
**Presence of symptoms** ^#^								
Cough	26	(100)	118	(86.8)	404	(88.2)	548	(88.4)
Fever	24	(92.3)	128	(94.1)	358	(78.2)	510	(82.3)
Shortness of breath	9	(34.6)	103	(75.7)	390	(85.2)	502	(81.0)
Myalgia	15	(57.7)	64	(47.1)	81	(17.7)	160	(25.8)
Chest pain	6	(23.1)	26	(19.1)	42	(9.2)	74	(11.9)
Sore throat	8	(30.8)	12	(8.8)	11	(2.4)	31	(5.0)
Running nose	0	(0)	1	(0.7)	10	(2.2)	11	(1.8)
Fatigue	3	(11.5)	31	(22.8)	68	(14.8)	102	(16.5)
Headache	12	(46.2)	42	(30.9)	149	(32.5)	203	(32.7)
Loss of appetite	3	(11.5)	35	(25.7)	90	(19.7)	128	(20.6)
Anosmia	4	(15.4)	31	(22.8)	45	(9.8)	80	(12.9)
Loss of taste	4	(15.4)	27	(19.9)	48	(10.5)	79	(12.7)
Diarrhea	2	(7.7)	31	(22.8)	36	(7.9)	69	(11.1)
Hemoptysis	0	(0)	5	(3.7)	16	(3.5)	21	(3.4)
**Comorbidities**								
Diabetes mellitus	4	(15.4)	14	(10.3)	77	(16.8)	95	(15.3)
Hypertension	5	(19.2)	23	(16.9)	123	(26.9)	151	(24.4)
COPD	0	(0)	1	(0.7)	28	(6.1)	29	(4.7)
Hypothyroidism	0	(0)	6	(4.4)	24	(5.2)	30	(4.8)
Asthma	0	(0)	2	(1.5)	1	(0.2)	3	(0.5)
PTB	0	(0)	1	(0.7)	9	(2.0)	10	(1.6)
None	20	(76.9)	98	(72.1)	262	(57.2)	380	(61.3)
**Saturation at admission**								
≥94%	24	(92.3)	43	(31.6)	0	(0)	67	(10.8)
86–93%	2	(7.7)	93	(68.4)	102	(22.3)	197	(31.8)
70–85%	0	(0)	0	(0)	231	(50.4)	231	(37.3)
<70%	0	(0)	0	(0)	121	(26.4)	121	(19.5)
Not available	0	(0)	0	(0)	4	(0.9)	4	(0.7)
**Duration from diagnosis to admission (in days)**								
≤0	4	(15.4)	38	(27.9)	132	(28.8)	174	(28.1)
1–3	12	(46.2)	50	(36.8)	155	(33.8)	217	(35.0)
4–7	9	(34.6)	34	(25.0)	122	(26.6)	165	(26.6)
8–14	1	(9.6)	13	(9.6)	38	(8.3)	52	(8.4)
≥15	0	(0)	1	(0.7)	11	(2.4)	12	(1.9)
**Vaccine status**								
Received	6	(23.1)	25	(81.6)	57	(12.5)	88	(14.2)
Not received	20	(76.9)	111	(18.4)	401	(87.5)	532	(85.8)

* Column percentages with total number in each group as denominator; ^#^ Symptoms were recorded at the time of presentation; ^ Severity was categorized according to WHO clinical living guideline; COVID-19: Coronavirus disease 2019; COPD: Chronic Obstructive Lung Disease; PTB: Pulmonary Tuberculosis.

**Table 3:** Uptake of laboratory investigations and their findings in COVID-19 patients (stratified by severity of illness) admitted in a tertiary teaching hospital of Nepal from April to September 2021.

Laboratory Parameters	Mild, N = 26				Moderate, N = 136				Severe/Critical, N = 458				Total, N = 620			
	Tested		Abnormal		Tested		Abnormal		Tested		Abnormal		Tested		Abnormal	
	*n*	(%) *	*N*	*(%) ^#^*	*N*	(%) *	*N*	*(%) ^#^*	*n*	(%) *	*n*	*(%) ^#^*	*n*	(%) *	*n*	*(%) ^#^*
Hemoglobin	25	(96.2)	0	(0)	127	(93.4)	2	(1.6)	430	(93.9)	15	(3.5)	582	(93.9)	17	(2.9)
Total leucocyte count	25	(96.2)	6	(24.0)	127	(93.4)	34	(26.8)	430	(93.9)	115	(26.7)	582	(93.9)	155	(26.6)
Platelet count	25	(96.2)	0	(0)	127	(93.4)	1	(0.8)	430	(93.9)	0	(0)	582	(93.9)	1	(0.2)
D-dimer	19	(73.1)	6	(31.6)	87	(64.0)	36	(41.4)	368	(80.3)	212	(57.6)	474	(76.5)	254	(53.6)
C-reactive protein	23	(88.5)	17	(73.9)	96	(70.6)	76	(79.2)	311	(67.9)	291	(93.6)	430	(69.4)	384	(89.3)
Random blood sugar	26	(100)	2	(7.7)	136	(100)	18	(13.2)	458	(100)	137	(29.9)	620	(100)	157	(25.3)
Serum creatinine	24	(92.3)	0	(0)	120	(88.2)	2	(0.2)	424	(92.6)	27	(6.3)	568	(91.6)	29	(5.1)
Blood culture	1	(3.8)	0	(0)	19	(14.0)	0	(0)	162	(35.4)	10	(6.2)	182	(29.4)	10	(5.5)
Sputum culture	1	(3.8)	0	(0)	20	(14.7)	4	(20.0)	168	(36.7)	44	(26.2)	189	(30.5)	48	(25.4)

* Percentages calculated with total number in each group as denominator; ^#^ Percentages calculated with number of patients tested as denominator. COVID-19: Coronavirus disease-19. Abnormal: Hemoglobin < 9 g/dL; Total Leucocyte Count < 4000 and >11,000 cells/cu mm; Platelet Count < 50,000 cells/cu mm; D-dimer > 500; C-reactive protein > 5 and positive; Random Blood Sugar > 200 mg/dL; Serum Creatinine > 1.2 mg/dL; Blood Culture—Bacterial growth found; Sputum Culture—Bacterial growth found.

**Table 4:** Treatment administered to COVID-19 patients (stratified by severity of illness at the time of presentation) admitted in a tertiary hospital of Nepal from April to September 2021.

**Treatment**	**Mild, N = 26**		**Moderate, N = 136**		**Severe/Critical, N = 458**		**Total, N = 620**	
	***n***	(%) *	***n***	(%) *	***n***	(%) *	***N***	(%) *
***Medication***								
Remdesivir	1	(3.8)	19	(14.0)	116	(25.3)	136	(21.9)
*Corticosteroids ^#^*	8	(30.8)	101	(74.3)	458	(100)	567	(91.5)
Dexamethasone	8	(30.8)	101	(74.3)	440	(96.1)	549	(88.5)
Methylprednisolone	0	(0)	3	(2.2)	67	(14.6)	70	(11.3)
*Anticoagulant agent ^$^*	11	(42.3)	106	(77.9)	458	(100)	575	(92.7)
UFH	8	(30.8)	84	(61.8)	399	(87.1)	491	(79.2)
Enoxaparin	0	(0)	8	(5.9)	214	(46.7)	222	(35.8)
Aspirin	10	(38.5)	60	(44.1)	322	(70.3)	392	(63.2)
Antibiotics	6	(23.1)	43	(31.6)	387	(84.5)	436	(70.3)
***Oxygen support***								
Invasive mechanical ventilation	0	(0)	1	(0.7)	7	(1.5)	8	(1.3)
Non-invasive ventilation	1	(3.8)	1	(0.7)	149	(32.5)	151	(24.4)
Low-flow O_2_ device	4	(15.4)	107	(78.7)	302	(66.0)	413	(66.6)
No support given	21	(80.8)	27	(19.9)	0	(0.0)	48	(7.7)
***Median days of oxygen support (IQR)***	0	(0)	2	(2)	7	(10)	5	(8)
***Median days of hospital stay (IQR)***	4	(2)	5	(3)	9	(10)	7	(7)

* Percentages calculated with total number in each group as denominator; ^#^ Used either dexamethasone or methyl prednisolone; ^$^ Used either UFH, enoxaparin or aspirin. Abbreviation: COVID-19: coronavirus disease 2019; UFH: unfractionated heparin; IQR: interquartile range.

## Data Availability

Data will be made available from the corresponding author on request.

## Open Access Statement and Disclaimer

In accordance with the WHO’s open-access publication policy for all work funded by WHO or authored/co-authored by WHO staff members, WHO retains the copyright of this publication through a Creative Commons Attribution IGO license (http://creativecommons.org/licenses/by/3.0/igo/legalcode, accessed on 12 November 2022) which permits unrestricted use, distribution and reproduction in any medium provided the original work is properly cited. There should be no suggestion that WHO endorses any specific organization, products or services. The views expressed in this article are those of the authors and do not necessarily reflect those of their affiliated institutions. The use of the WHO logo is not permitted. This notice should be preserved along with the article’s original URL.
